# Gold Nanoparticles Crossing Blood-Brain Barrier Prevent HSV-1 Infection and Reduce Herpes Associated Amyloid-βsecretion

**DOI:** 10.3390/jcm9010155

**Published:** 2020-01-07

**Authors:** Rodriguez-Izquierdo I, Serramia MJ, Gomez R, De La Mata FJ, Bullido MJ, Muñoz-Fernández MA

**Affiliations:** 1 Head Immunology Section. Laboratorio InmunoBiología Molecular, Hospital General Universitario Gregorio Marañón (HGUGM), 28007 Madrid, Spain; igna.iri.93@gmail.com (R.-I.I.);; 2 Instituto de Investigación Sanitaria Gregorio Marañón (IiSGM), 28007 Madrid, Spain; 3 Spanish HIV-HGM BioBank, 28007 Madrid, Spain; 4 Departamento Química Orgánica e Inorgánica, Instituto de Investigación Química “Andrés M. del Río” (IQAR), Universidad de Alcalá (UAH), 28805 Alcalá de Henares, Spain; rafael.gomez@uah.es (G.R.);; 5 Instituto Ramón y Cajal de Investigación Sanitaria (IRYCIS), 28034 Madrid, Spain; 6 Networking Research Center on Bioengineering, Biomaterials and Nanomedicine (CIBER-BBN), 28029 Madrid, Spain; 7 Departamento de Biología Molecular, Centro de Biología Molecular Severo Ochoa (CSIC-UAM), Universidad Autónoma de Madrid, 28049 Madrid, Spain; mjbullido@cbm.csic.es; 8 Centro de Investigación Biomédica en Red sobre Enfermedades Neurodegenerativas (CIBERNED), 28031 Madrid, Spain

**Keywords:** HSV-1, gold nanoparticles, central nervous system, amyloid-β peptides, neurodegeneration

## Abstract

Infections caused by HSV-1 and their typical outbreaks invading the nervous system have been related to neurodegenerative diseases. HSV-1 infection may deregulate the balance between the amyloidogenic and non-amyloidogenic pathways, raising the accumulation of amyloid-β peptides, one of the hallmarks in the neurodegenerative diseases. An effective treatment against both, HSV-1 infections and neurodegeneration, is a major therapeutic target. Therefore, gold nanoparticles (NPAus) have been previously studied in immunotherapy, cancer and cellular disruptions with very promising results. Our study demonstrates that a new NPAus family inhibits the HSV-1 infection in a neural-derived SK-N-MC cell line model and that this new NPAus reduces the HSV-1-induced β-secretase activity, as well as amyloid-β accumulation in SK-APP-D1 modifies cell line. We demonstrated that NPAuG3-S8 crosses the blood-brain barrier (BBB) and does not generate cerebral damage to in vivo CD1 mice model. The NPAuG3-S8 could be a promising treatment against neuronal HSV-1 infections and neuronal disorders related to the Aβ peptides.

## 1. Introduction

Herpes Simplex Virus (HSV) type 1 is one of the most common infections in the human population nowadays [1]. More than 65% of the human population up to 50 years is infected by HSV-1. This virus establishes lifelong latent infections within the nervous system [2], avoiding the immune system mechanisms and raising the subsequent recurrent episodes [3]. In serious cases, the damage could cause corneal disease or encephalitis. Although the herpes simplex virus encephalitis (HSE) can result from primary infection as well as for recurrent HSV-1 episodes, HSV-1 impacts especially in immunocompromised patients, increasing the risk of developing HSE [4]. Once the primary infection takes place, the HSV-1 invades the peripheral nervous system (PNS), mainly sensory neurons, and generates a latent infection in trigeminal ganglia [3], where the virus is undetectable [5,6]. HSV-1 has been related to various neurodegenerative disorders in the last decade [7], and novel studies have contributed to new data and valuable approaches to understand the mechanisms of neurocognitive pathology. One of the most frequent HSV-1 infection-associated diseases is Alzheimer’s disease (AD) [8,9,10,11,12,13]. Life expectancy after diagnosis varies between 7 and 15 years, depending on the severity at the time of diagnosis. As familial AD (FAD), AD is caused by mutations in three genes which codify the amyloid precursor protein (APP) [14], whereas sporadic AD (SAD) is linked to several genetic and environmental risk factors, consistently related to neural infections such as those caused by HSV-1 [11,12,13,15,16].

FAD and SAD have common neuropathological hallmarks, mainly the presence of senile plaques and neurofibrillary tangles [17]. The senile plaques are generated by extracellular accumulation of amyloid-β peptide (Aβ) [18]. Those Aβ plaques are the result of deregulation in the balance between amyloidogenic and non-amyloidogenic pathways of APP [19,20,21]. In both routes, the APP transmembrane protein is firstly proteolyzed by α or β-secretase, followed by the action of the γ-secretase [19]. When the regulation between both pathways is affected, Aβ peptides will accumulate and remain as intracellular soluble oligomers and extracellular senile plaques [19,22,23].

HSV-1 induced the intracellular accumulation of Aβ by the inhibition of the non-amyloidogenic pathway [24,25]. This infection also modifies the proteolytic processing of the APP [26], as well as the autophagy process [24]. These results show a potential correlation between AD and HSV-1 [11,12,13,27], revealing the interactions amidst the HSV-1 infections in the nervous system and the higher risk to develop neurodegenerative disorders. 

These results show a correlation between AD patients and HSV-1, where the presence of viral DNA was much more frequent in AD patients than in healthy individuals [27], revealing the interactions amidst the HSV-1 infections in the nervous system and the higher risk of developing AD. In agreement, the presence of HSV-1 in certain locations of the nervous system can be related to morphological changes shown in the neurons, related to the development of several neurodegenerative diseases such as AD [27].

The therapy against HSV-1 is based on the use of nucleotide analogous drugs such as acyclovir (ACV), famciclovir (FAM) or valacyclovir (VCV) being their main target the viral DNA polymerase. However, those drugs only mitigate and modulate the HSV-1 symptoms [28], but they are not capable of eliminating the HSV-1 latency [28,29]. ACV, the most used anti-HSV-1 drug, generates resistance in immunocompetent patients with recurrent episodes [30,31,32]. This limitation highlights the need to develop new anti-HSV-1 compounds based on alternative mechanisms of action. A widespread kind of nanoparticles (NPs) have shown great results inhibiting several steps of the viral cycle in HSV-1 and HIV-2 infection [33,34,35], as well as in other latent viruses [36,37,38]. They have a controlled synthesis consisting of a core with several sequential branching units, which present several end-groups [39]. The capability to synthesize this hyperbranched polymer with different cores or functional groups provides these NPs a versatile functionalization.

Therefore, the objective of the present study is to evaluate the anti-HSV-1 activity of a new NPAu family, as well as to elucidate the mechanism of action of this inhibition and evaluate the biocompatibility of these new nanoparticles in an *in vivo* model. 

## 2. Materials and Method

### 2.1. Cell Lines, Culture Conditions and Virus 

Human neuroepithelioma cell line SK-N-MC (ATTC HTB-10, ATCC, Manassas, Virginia, USA), and African green monkey kidney Vero cell line (ATTC CCL-81, ATCC, Manassas, Virginia, USA) were obtained from the American Type Culture Collection. Stable transfected SK-N-MC amyloid precursor protein (SK-APP-D1, these cells were generated from SK-N-MC cells (ATCC HTB10, Manassas, Virginia, USA) modified to stably expressing the human isoform APP695 and these cells were kindly provided by Dra. MJ. Bullido (Centro Biología Molecular Severo Ochoa, CBMSO, Madrid, Spain).

SK-N-MC and SK-N-MC APP-D1 cell lines were grown in Dulbecco’s Modified Eagle’s Medium (DMEM; Biochrom AG, Berlin, Germany) supplemented with 10% fetal bovine serum (FBS); Vero cell line was grown in DMEM supplemented with 5% FBS. All culture mediums contain 1% L-glutamine, and an antibiotic mix (125 μg/mL ampicillin, 125 μg/mL cloxacillin and 40 μg/mL gentamicin); (Sigma, St. Louis, Missouri, USA). All cell lines were cultivated in 5% CO_2_ at 37 °C.

HSV-1 strain Kos 1.1 was kindly provided by Dra. MJ. Bullido and expanded on the Vero cell line, titrated by plaque assay and stored at −80 °C. 

### 2.2. Nanoparticles

**Figure 1:**
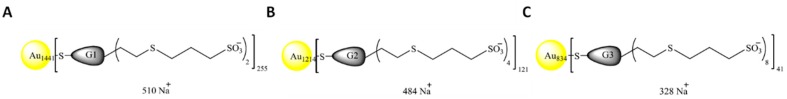
Schematic representation of Gold nanoparticles (NPs). NPs decorated with (**A**) first generation dendron with two sulfonate end groups, (**B**) second generation dendron with four sulfonate end groups and (**C**) third generation dendron with eight sulfonate end groups. The generation of NPs is determined by considering that each generation corresponds to the number of repeating layers of silicon atoms forming the NP.

NPs were synthesized according to described procedures by Bermejo et al [39]. The gold NPAuG1-S2, NPAuG2-S4, and NPAuG3-S8 were selected according to the generation number, type and number of peripherical charges and the focal point. The three selected NPAus were briefly described in Table 1 and the schematic structures of the polyanionic carbosilane NPAus were represented in Figure 1. NPs were dissolved in distilled water (Promega, Madrid, Spain) as well as subsequent dilutions to working concentrations.

### 2.3. Cell Viability Assay

Cellular mitochondrial metabolism was evaluated by MTT assay. This assay is based on the reduction of the 3-(4,5-dimethylthiazol-2-yl)-2,5-diphenyl-tetrazoliumbromide (MTT) (Sigma, St. Louis, Missouri, USA) to formazan crystals as an indicator of dendrimer-induced toxicity. SK-N-MC cell line was seeded in 96-well plates at 5×10^5^ cells/well. Cells were exposed to a range of concentrations (10–500 nM) for each NP. After 24 h, SK-N-MC cells were incubated with 0.5 mg/mL MTT for 2 h at 37 °C, and MTT/formazan crystals were dissolved with DMSO (Honeywell, Charlotte, North Carolina, USA). Optical densities were measured at 570/690 nm (Synergy 4 microplate reader, BioteK, Winooski, Vermont, USA). DMSO was used as death control.

### 2.4. HSV-1 Infection

SK-N-MC cell line was seeded in 12-well plates (6×10^5^ cells/well), and exposed to the maximum non-toxic concentration of each NP for 1 h and HSV-1 infected at a multiplicity of infection (MOI) of 1 plaque-forming unit (pfu)/cells for 1 h in the case of pre-treatment assay. For treatment assays, SK-N-MC cell line was treated and infected simultaneously at MOI 1 for 1 h. At 24 h post-infection, serially diluted supernatants were titrated by plaque assay on Vero cell line, previously seeded in 12-well plates (3.75×10^5^ cells/well). Vero cells were infected with 350 µL supernatants at seriated dilutions and washed with PBS at 2 h post-infection to remove unabsorbed viruses. HSV-1 infection remained in DMEM containing 2% FCS and 0.4% IgG. After 72 h, the medium was removed, Vero cells were stained with Methylene Blue 300 mg/L (Sigma, St. Louis, Missouri, USA) 1 h, and the lysis plaques were counted. Results were related to infection controls.

### 2.5. Cellular Protection against HSV-1 Infection

SK-N-MC cell line was 6×10^5^ cells/well seeded in 12-well plates and exposed to the inhibitory concentration for 1 h. SK-N-MC cells were washed to remove any excess of unbound NPs and the HSV-1 infection was performed at MOI 1, 1 h at 37 °C. Supernatants were titrated 24 h post-infection on Vero cells by plaque assay as described before.

### 2.6. Interactions Nanoparticles-HSV-1

HSV-1 (MOI 1) was incubated 1 h with the inhibitory concentration of each NP at 4 °C in an eppendorf. Vero cells were seeded at 3x10^5^ cells/well in a 12-well plate and exposed to the HSV-1-NPs mix, 1 h at 37 °C. Supernatants were titrated 48 h post-infection by plaque assay as described before.

### 2.7. β-Secretase Activity

SK-APP-D1 cells (5×10^6^) were treated with NPs and HSV-1 infected at MOI 1 for 1h. Mock HSV-1 infected control was made with virus-free suspension. After 3 h post-HSV-1 infection, β–secretase activity was determined using commercial kits (BioVision, Milpitas, California, USA) according to the manufacturer’s instructions. Plates were incubated in dark at 37 °C for 1 h, and fluorescence recorded at 420 mn using a microplate reader (Synergy 4 microplate reader, BioteK, Winooski, Vermont, USA). The activity was related to non-treated cells.

### 2.8. Measurement of Secreted Aβ

The secreted Aβ was measurement by an enzyme-linked immunosorbent assay (Kit ELISA, Thermo Fisher scientific, Spain). The SK-APP-D1 cells were mock-infected or HSV-1 infected. The supernatants were assayed for human Aβ40 and Aβ42 using a commercial sandwich ELISA kit (Wako, Osaka, Japan) according to the manufacturer’s instructions. The sensitivity of both kits is 1.0–100.0 pmol/L and 0.1–20.0 pmol/L, respectively. Related to the specificity, both kits present ≦0.1 of cross-reactivity with others Aβ. The standard curve was performed using 100 pmol/L of Aβ40 or 20 pmol/L Aβ42 stock solution. 

### 2.9. Histological Studies in CD1 Mice 

Histological studies in mice brains were evaluated by hematoxylin-eosin staining. Twenty four CD1 mice 8 weeks old, 22 ± 3 g (Charles River Laboratories, Wilmington, MA, USA) were purchased and housed in CBMSO animal husbandry. Mice studies were conducted and approved by the CBMSO Institutional Animal Care and Use Committee (CEEA-CBMSO, Madrid, Spain). One mouse control group treated with PBS and three mice groups treated with gold NPAuG3-S8 at 10 h, 24 h and 48 h were established for intravenous NP administration. Twenty-one of the twenty four mice were injected intravenously in the tail vein with 3.5 mg/Kg of NPAuG3-S8 previously dissolved in PBS and 3 mice were injected only in PBS as control.

Mice were sacrificed at 10 h, 24 h and 48 h after the inoculation. Then, brains were extracted and fixed on PFA 4%. Samples were included in paraffin by passing through increasing alcohol, two xylol baths, and one paraffin bath. Subsequently, the brains were cut by means of a microtome and processed for hematoxylin and eosin. Brain sections were finally mounted with DPX. The brains were carved making three cuts to make the assessment of the different parts of it. The assigned values (score) were 0 (no changes): when no injuries were observed or the changes observed were within normality; 1 (minimum): when the changes were scarce but exceeded those considered normal; 2 (slight): the lesions were identifiable but with moderate severity; 3 (moderate): important injuries but can still increase in severity; and 4 (very serious): very serious injuries. 

### 2.10. Permeability of Blood-Brain Barrier to NPAuG3-S8 in CD1 Mice

To determine the presence of NPAuG3-S8 in the brains of CD1 mice, the same four randomized CD1 mice groups were used. Mice were injected intravenously in the tail vein with 3.5 mg/Kg of NPAuG3-S8 dissolved in PBS and mice were sacrificed at 10 h, 24 h and 48 h after the inoculation. Then, the brain of each mouse was extracted and was flashed frozen in Tissue-Tek O.C.T. (Sakura Finetek, Torrance, California, USA). 

For cryogenic sections, slides with histological sections were incubated in 0.1% Triton X-100 for 5 min and washed. NPAuG3-S8 was stained by the silver enhancement kit (Sigma, St. Louis, Missouri, USA) for 5 min, washed and fixed with 2.5% sodium thiosulfate solution (STS). Counterstain with eosin and mounts of histological sections were performed in order to determine the presence of NP by light microscopy.

### 2.11. Statistical Analysis

Statistical analysis including mean and standard deviation (SD), or median and interquartile ranges were analyzed with GraphPad Prism v5.0 software (GraphPad, San Diego, CA, USA) using non-parametric unpaired *t*-Test. Differences were considered significant at *p* < 0.05 (*), *p* < 0.01 (**), or *p* < 0.001 (***). All dates were obtained of 2 or 3 independent experiments performed by duplicate or triplicate.

## 3. Results

### 3.1. Cytotoxicity of Nanoparticles

The cytotoxicity of NPAuG1-S2, NPAuG2-S4, and NPAuG3-S8 was evaluated in human neuroepithelioma SK-N-MC cell line, using a concentration rate for each NPAu from 10 to 500 nM. SK-N-MC cells were treated for 24 h with increasing concentrations of NPs, which were considered toxic when the survival rate was <80%. 10µM dextran and 10% DMSO were used as non-treated, harmless and cell death controls, respectively.

**Figure 2:**
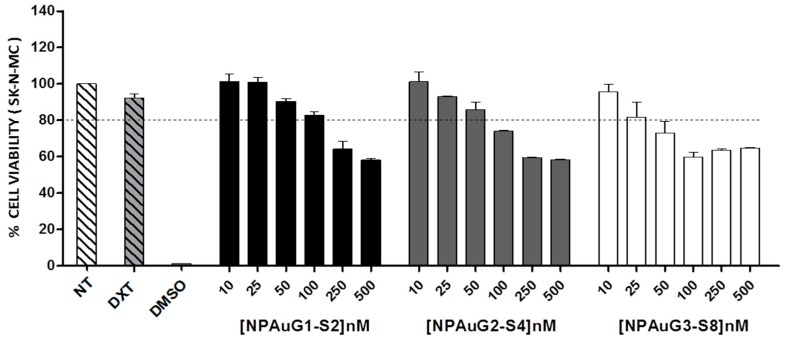
Cytotoxicity associated with NPAus in SK-N-MC human neuroepithelioma cell lines at 24 h post-treatment using MTT assay. SK-N-MC cells were treated with increasing concentrations of gold NPs from 10 nM to 500 nM. SK-N-MC cells were not treated as control of viability or treated with 10% of DMSO as control of cell death. The percent of cell viability was calculated as optical density. The 80% of viability was set asa limit of toxicity. Data are represented as mean ± SD of three experiments performed in triplicate. Abbreviations: NT= non-treated; DXT= Dextran; DMSO= dymethyl sulfoxide.

The MTT results reveal that NPAuG3-S8 was non-toxic at 25 nM, NPAuG2-S4 at 50 nM, and NPAuG1-S2 at 100 nM (Figure 2). 

### 3.2. Anti-HSV-1 Activity of Nanoparticles

We further studied the antiviral activity of NPAus against HSV-1 in SK-N-MC cells under pre- and treatment conditions. Each NP was assayed at maximum non-toxic concentrations. ACV (20 μM) was used as a positive control for HSV-1 inhibition. SK-N-MC cells were pre-treated with NP for 1 h and infected or simultaneously treated and infected with HSV-1at an MOI 1. 

**Figure 3:**
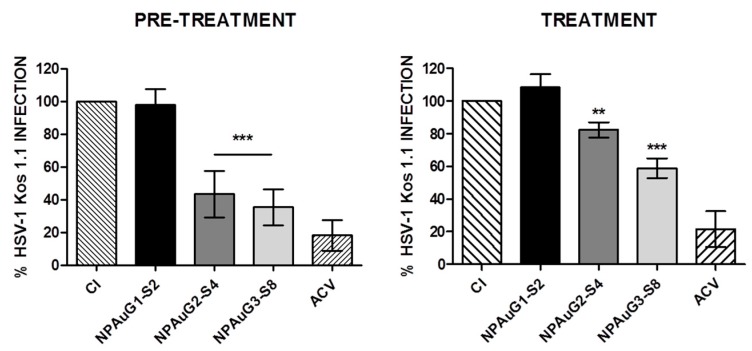
Inhibition of HSV-1 infection by NPAus in SK-N-MC cell lines. SK-N-MC cell line was exposed to the maximum non-toxic concentration of each NPAus and HSV-1 infected at MOI 1 for 24 h in pre-treatment assay or 1h in treatment assay. 20 μM ACV was used as a positive control of HSV-1 inhibition. Data represent mean ± SD from at least three independent experiments performed by duplicate. **: *p* < 0.01; ***: *p* < 0.001. CI: HSV-1 infection at MOI 1.

HSV-1 infection titration was measured by plaque assays on Vero cells. The pre-treatment of SK-N-MC cells with NPAuG2-S4 and NPAuG3-S8 inhibited the HSV-1 infection by 56.5% and 64.5%, respectively (*p* < 0.01) (Figure 3). However, the treatment of NPAuG1-S2, NPAuG2-S4, and NPAuG3-S8 showed an inhibition up to 20% (*p* < 0.05), 25% (*p* < 0.05), and 40%(*p* < 0.001), respectively (Figure 3) in SK-N-MC cells.

Therefore, NPAuG2-S4 and NPAuG3-S8 with high activity against HSV-1 infection were selected for new studies.

### 3.3. Gold Nanoparticles Inhibit HSV-1 Infection through Cellular Protection

**Figure 4:**
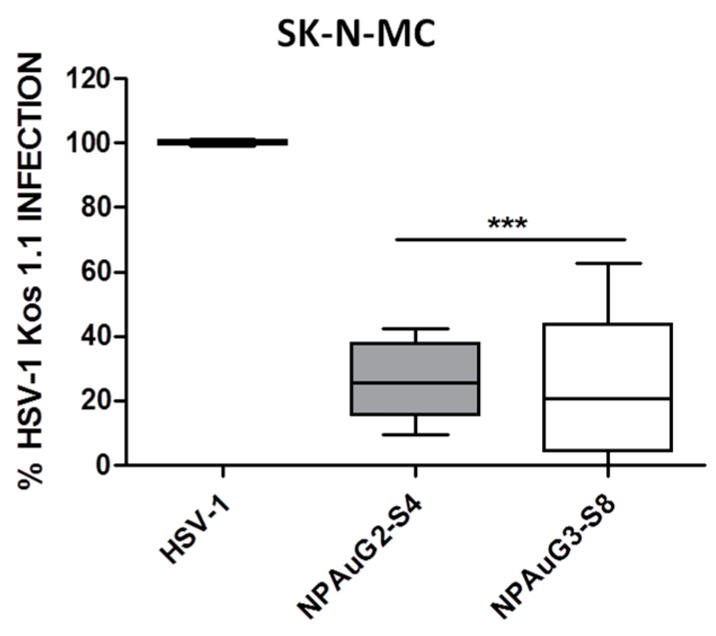
Cellular protection mediated by NPAus. SK-N-MC cell line was exposed to the maximum non-toxic concentration for 1 h. SK-N-MC cells were washed to eliminate the NPs and infected at MOI 1 for 1 h. Data represent median and interquartile range ± SD from three independent experiments performed by duplicate. ***: *p* < 0,001; HSV-1 infection control at MOI 1.

Inhibition at the level of viral entry may be due to the binding of the NPs either to cellular or viral receptors, resulting in both cases in the blockade of the binding for the virus to the cell. To evaluate the percentage of HSV-1 inhibition due to the interaction of the NPAus with cellular receptors, acting as competitors of HSV-1 binding sites, SK-N-MC cells were pre-incubated 1 h at 37 °C with NPAuG2-S4 or NPAuG3-S8. SK-N-MC cells were washed to remove the unbound NPs to avoid their interaction with HSV-1. The HSV-1 infection was measured by plaque assays on Vero cells. Interestingly, the SK-N-MC cells showed significant cellular protection against HSV-1 infection by NPAuG2-S4 (75%) and NPAuG3-S8 (74%) (Figure 4).

### 3.4. Gold Nanoparticles Inhibit HSV-1 Infection through Viral Action

**Figure 5:**
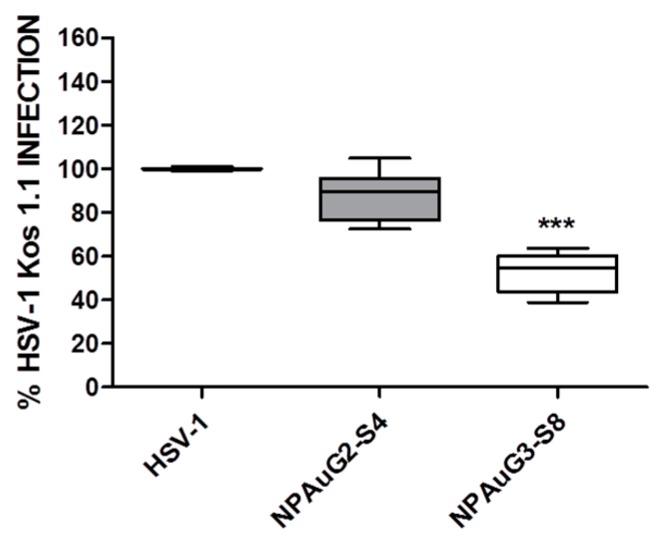
Activity against HSV-1. NPAus were incubated with MOI 1 of HSV-1 at 4 °C. After 1 h, Vero cells were infected with HSV-1-NP mix, and the infection was revealed by plaque assay as performed before. Data represent median and interquartile range ± SD from four independent experiments performed by duplicate. ***: *p* < 0.001; HSV-1 infection control at MOI 1.

To evaluate the percentage of inhibition due to the interaction of the NPAus with HSV-1, second and third selected NPs were incubated with MOI 1 of HSV-1 1 h at 4ºC. The incubation was performed at 4ºC with the objective to prevent the loss of infectivity. This experiment was performed on Vero cells due to the fact that the aim was independent of the cells used, in order to evaluate the activity of the NPs against HSV-1. Vero cells were incubated with NPAus-HSV-1 1h at 37ºC and infection was revealed by plaque assay. The two selected NPAus inhibited HSV-1 infection in a significant manner by interacting with HSV-1. Our data showed that NPAuG2-S4 inhibited the viral infection acting against HSV-1 by 20% and NPAuG3-S8 by approximately 50% (Figure 5), showing that NPs interacted with the viral particles and prevented the HSV-1 infection.

### 3.5. Nanoparticles Revert the β-Secretase Activity Increase Induced by HSV-1 to Basal Levels

**Figure 6:**
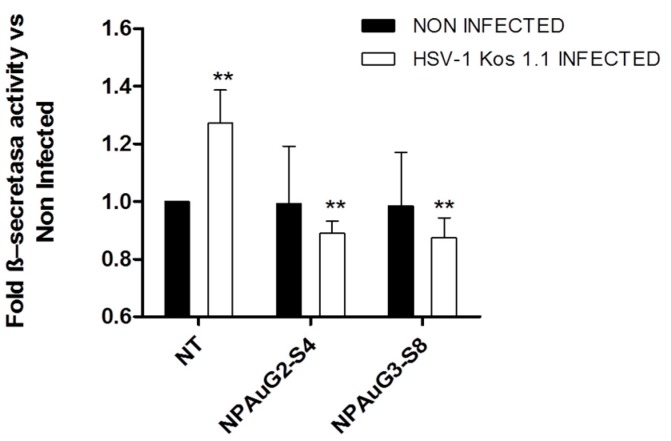
Levels of β-secretase activity in the SK-APP-D1cell line. SK-APP-D1cells were treated with NPAus, or NPAus infected with HSV-1. Data represent mean ± SD of three independent experiments performed by triplicate. **: *p* < 0.01; NT: SK-APP-D1 cells. Statistical analysis vs SK-APP-D1 non-treated control cells.

To verify the influence of NPAus on APP processing, the β-secretase activity was determined using a specific kit (BioVision, Milpitas, California, USA). Thus, 5×10^6^ SK-APP-D1 cells were treated with NPAus to evaluate the NP activity on β-secretase, or treated with NP and infected with HSV-1. The HSV-1 infection increased in a significant manner in the β-secretase activity (Figure 6), favoring the amiloidogenic pathway, and raising the probability of Aβ aggregates appearance. Interestingly enough, both NPs were able to reduce the increase due to HSV-1 in the β-secretase activity to non-treated levels, even when SK-APP-D1 cells were pre-treated and HSV-1 infected.

Interestingly, NPAus appeared to decrease the β-secretase activity in HSV-1 infected SK-APP-D1 cells to even below the levels of non-infected cells, suggesting that these NPAus have the potential to act against HSV-1 infection and even more, as a new potential treatment in the Aβ HSV-1 related neuronal disorders.

### 3.6. Effect of Nanoparticles on Secreted Aβ

It is widely accepted that HSV-1 infection of neuronal cells induces intracellular amyloid accumulation that is associated with the appearance of amyloid plaques in the mouse model and in human brain samples. The Aβ 1-40 (Aβ40) and Aβ 1-42 (Aβ42) peptides are cleaved from the APP by the sequential action of β- and γ-secretase. Aβ42 is more prone to aggregate than Aβ40. The initial deposition in the Aβ plaques formation is due to Aβ42, although Aβ40 appears in the advanced stages of neurodegenerative disorders such as AD.

**Figure 7:**
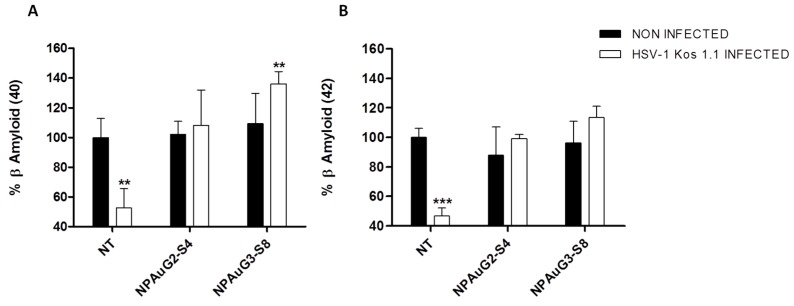
Secreted Aβ40 and Aβ42 levels on SK-APP-D1 cells. SK-APP-D1 cells were treated with NPAuG2-S4 or NAPuG3-S8 and non-infected (**A**) or infected with HSV-1 (**B**) at MOI 1, 1 h at 37 °C. Quantitative analysis of extracellular amyloid-β 1-40 or Aβ40 and 1-42 or Aβ42 levels were made by ELISA. Data represent mean ± SD of at least three independent experiments performed by duplicate. ***: *p* < 0.001; **: *p* < 0.01; NT: SK-APP-D1 cells. Statistical analysis vs. NT SK-APP-D1 non-treated cell control.

In order to evaluate if NPAus modify the accumulation and secretion of Aβ, SK-APP-D1 cells were treated with NPAus and infected with HSV-1 at MOI 1. The supernatants were analyzed by ELISA assay for Aβ40 and Aβ42 (Wako, Osaka, Japan). The HSV-1 infection decreased the secreted Aβ40, as was previously described [26], and both NPAus recovered those values up to non-treated data (Figure 7A). Interestingly, NPAuG3-S8 potentiated the secretion of Aβ40 (Figure 7A) being, once more, the most efficient NPAus in blocking the toxic Aβ accumulation as we previously showed in Figure 6. The same behavior was showed for Aβ42. Both studied NPAus were able to recover the normal values of secreted Aβ42, once SK-APP-D1 cells were HSV-1 infected (Figure 7B). Once more, NPAuG3-S8 showed the best results. It is important to note that NPAuG3-S8 could be considered as a new potential treatment in the HSV-1AD-related context.

### 3.7. Histological Studies in CD1 Mice

NPAuG3-S8 was selected for in vivo studies in CD1 mice. The objective was to evaluate the tissue damage in the CD1 mouse brain. The histopathological study was made with samples of 21 brains from 21 two-month-old CD1 mice treated with intravenous NPAuG3-S8. The sacrifices were made at 10 h, 24 h and 48 h after the inoculation with NPAuG3-S8. The results obtained from the 21 brains of CD1 mice previously treated with NPAuG3-S8 were compared with the results obtained from the 3 brains of CD1 mice previously treated with PBS. The toxicity of NPAuG3-S8 was measured by the presence of inflammatory infiltrates, which in the case of the brain is characterized by infiltration of the microglia cells, microglial satellitation, or presence of the microglia cells surrounding neuronal bodies, presence of vacuoles inside the neuronal cytoplasm, the existence of vascular lesions such as hemorrhage and edema, and finally, the presence of necrosis or malacia. The signs were scored as 0 (no change); 1 (minimum change); 2 (light change); 3 (moderate change) and 4 (very serious).

**Figure 8:**
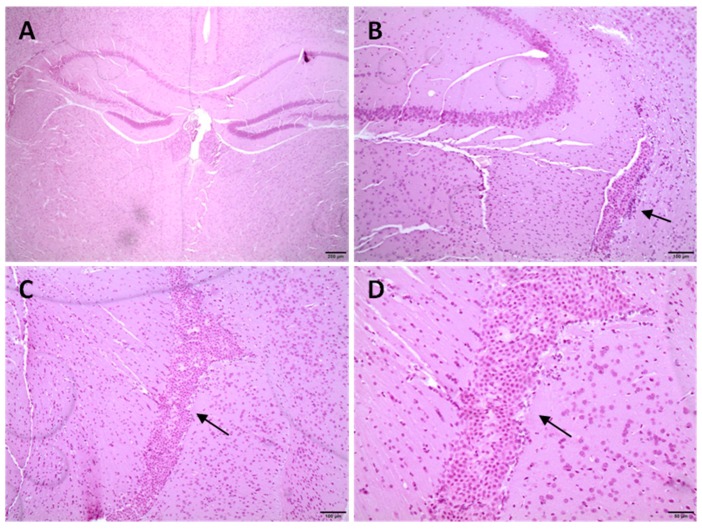
Histological sections of diencephalon midbrain. Cerebral samples were cut by means of a microtome and processed for hematoxylin and eosin. (**A**,**B**) Diencephalon sections of 24 h post-treatment (mouse 14). Minimal proliferation of the choroidal plexus epithelium (arrow). (**C**,**D**) Midbrain sections of 48 h post-treatment (mouse 23). Moderate proliferation of the epithelium of the choroid plexuses that occupy the lumen of one of the ventricles (arrows).

The histological studies at 10 h, 24 h or 48 h post-inoculation of NPAuG3-S8 in the mice brains showed that the hemorrhage, edema or presence of areas of necrosis was not detected (Table 2). Moreover, when CD1 mice were treated with NPAuG3-S8 and analyzed 10 h, 24 h or 48 h post-inoculation, no damage or alteration onto the brain epithelium was detected. Only a minimal or moderate proliferation of the epithelium of the choroid plexuses was detected in two mice, one at 24 h and other at 48 h (Figure 8). Our data suggest that NPAuG3-S8 could be a possible treatment against HSV-1 infection in the brain of mice. However, brain injuries had not been monitored after 48 h, and this fact could be a limitation that must be elucidated in futures studies. 

### 3.8. Capability of NPAuG3-S8 Crossing the Blood-Brain Barrier (BBB)

Once suggested that NPAuG3-S8 does not generate cerebral toxicity or tissue damage, we study the capability of NPAuG3-S8 to cross the BBB, with the objective to evaluate whether previous data were due to the fact that NPAuG3-S8 had a non-toxic effect or whether NPAuG3-S8 was unable to cross the BBB and consequently, this NPAuG3-S8 had no effect on the CNS. With the aim to study this possible hypothesis, CD1 mice were tail vein injected with NPAuG3-S8 and sacrificed at 10 h, 24 h and 48 h after the inoculation in the same way as in the histological study. The histological sections were stained with Silver Enhancement Kit (Sigma, Missouri, USA) for 5 min to determine the presence of NPAuG3-S8, and counterstained with eosin in order to decrease the contrast of staining.

**Figure 9:**
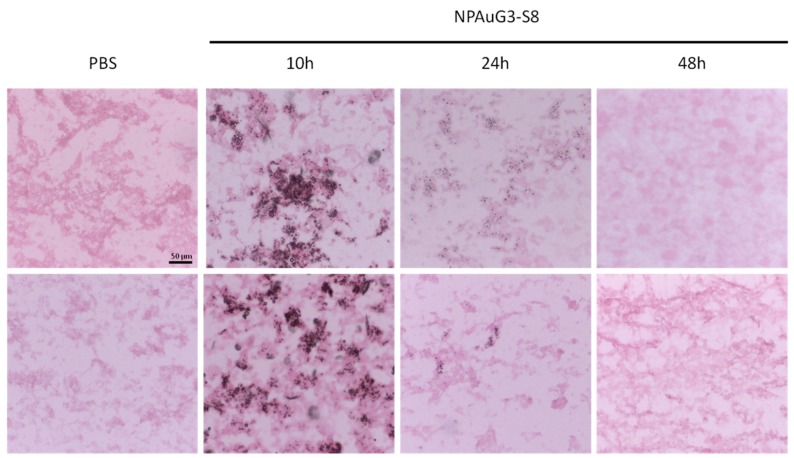
NPAuG3-S8 accumulation in brain tissue. Representative images for the presence of NPAuG3-S8 in brain tissue. The images show that NPAuG3-S8 at 10 h and 24 h cross the (blood-brain barrier) BBB and at 48 h it does not cross the BBB stained with silver enhancement and counterstained with eosin. PBS = Negative control of silver enhancement stain. All images were obtained at the same size as the scale bar shown.

The NPAuG3-S8 was detectable in brain tissue after 10 h post-inoculation, indicating clearly that this NPAuG3-S8 cross the BBB (Figure 9). However, 24 h post-inoculation the NPAuG3-S8 accumulation decreased (Figure 9).

Based on the previous results where NPAuG3-S8 seems to not have histological lesions in mice brain, and that NPAuG3-S8 cross the BBB, this NPAuG3-S8 could be a possible candidate for the treatment of HSV-1 infections in the CNS.

## 4. Discussion

In the last decade, researchers have provided increasing evidence of a potential relationship between HSV-1 and neurodegenerative disorders such as AD [10,11,12,13]. The fact that HSV-1 infection is related to AD can be due to the distinguishing characteristics of HSV-1 to establish latency in neurons [9]. Although HSV-1 infection is generally limited or asymptomatic, there is a percentage of HSV-1 infections that generate encephalitis or meningoencephalitis, which cause important lesions and brain alterations. Previous studies have related the HSV-1 infection with a high risk of developing senile plaques, neurofibrillary tangles, and AD [8,10]. New drugs that approach the treatment of HSV-1 from other strategies, such as acting in other points of the viral cycle or mechanisms of action are being researched. In this context, the NPs have shown their inhibitory action against HSV, HIV or Hepatitis [33,35,40,41]. Thus, NPAuG1-S2, NPAuG2-S4, and NPAuG3-S8 were assayed against HSV-1 in neuronal cell lines. Our data provide the first approach to understand the behavior of these NPs in the CNS.

Once the non-toxic concentrations of NPAuG1-S2, NPAuG2-S4, and NPAuG3-S8 were selected, the HSV-1 inhibition assays showed that pre-treatment with NPAuG2-S4 and NPAuG3-S8 have >50% of HSV-1 inhibitory activity, and 20% and 40% respectively in the treatment assay. The NPAuG1-S2 has no effect against HSV-1 infection at any condition tested.

Both NPAuG2-S4 and NPAuG3-S8 could be acting against the HSV-1 infection, interacting with some peripheral glycoprotein and preventing the HSV-1 infection, or binding to the host cell, occupying the places for the viral union, and preventing, in the same way, the HSV-1 infection. To analyze which mechanism is used by NPAuG2-S4 and NPAuG3-S8, the cellular protection mediated by NPs in SK-N-MC cells was analyzed. NPAuG2-S4 and NPAuG3-S8 significantly protected SK-N-MC cells (>70% and >75%, respectively) against HSV-1 infection. 

Thus, we studied whether these NPs act against HSV-1. Data showed that NPs present a significant neutralizing activity against HSV-1, with NPAuG3-S8 being the best one inhibiting HSV-1 infection. The main histopathological markers in AD are the neurofibrillary tangles and the amyloid-β accumulations. Related to the Aβ, the accumulation of Aβ40 and mainly Aβ42 are considered one of the hallmarks of early stages in AD [42]. AD etiology studies had described that initially the amyloid plaques are made by Aβ42 peptide, whereas Aβ40 is also present in plaques in more advanced stages of AD pathology. The relationship between NPs and the proteolytic pathway of APP, and, more interestingly, to the Aβ40 and Aβ42 accumulation becomes very important.

The interaction between HSV-1 infection and an over-accumulation of Aβ40 and Aβ42 peptides, as well as the deregulation in the amyloidogenic and non-amyloidogenic pathways, have been previously described [8,22,26,27,43]. To analyze the interactions between HSV-1 and AD-like markers and the NPAuG2-S4 and NPAuG3-S8 influence on those mechanisms, firstly the β-secretase activity was evaluated. The amyloidogenic pathway is mediated by the β-secretase activity which initiates that pathway and subsequently provokes the accumulation of Aβ peptides. Our data showed that NPAuG2-S4 and NPAuG3-S8 reduced the HSV-1-mediated increase of β-secretase activity to normal levels. Moreover, both NPs reduced these levels even below the control levels. The fact that NPAuG3-S8 reduces significantly the increase shown due to HSV-1 infection makes this NPAuG3-S8 a promising candidate for treatment against Aβ HSV-1 neuronal disorders.

We studied the ability of NPAuG2-S4 and NPAuG3-S8 to modify the accumulation in Aβ peptides induced by HSV-1 infection. Our results clearly showed that NPAuG2-S4 and especially NPAuG3-S8 were able to reduce the increase in β-secretase activity due to the HSV-1 infection. Based on our results, the last step was to evaluate the ability of NPAuG3-S8 to cross the BBB, in order to determine if this NPAuG3-S8 could be used as a possible treatment in the CNS. Our data reveals that NPAuG3-S8 crosses the BBB in brain mice and even more NPAuG3-S8 seems to be non-toxic for the brain tissue at 48 h. The lack of monitoring of brain injuries after 48 hours could be a limitation, however, based on the data obtained at 10, 24 and 48 h all data indicates that this NPAus could be non-toxic in the mice brain. This NPAuG3-S8 could be a possible candidate against HSV-1 infections in the CNS as well as a future treatment against other neurodegenerative diseases associated with amyloid peptides.

## 5. Conclusions

Our study reveals the biosafety of three gold NPs in SK-N-MC neural-derived cell lines. NPAuG2-S4 and NPAuG3-S8 are capable to inhibit the HSV-1 infection in neuronal cell lines, both in pre-treatment and treatment assays, rising the more significant inhibition rates in the treatment assays. We also showed that both NPAuG2-S4 and NPAuG3-S8 inhibit HSV-1 bycellular protection against the infection in SK-N-MC, and that NPAuG3-S8 has a dual behavior, acting against the virus as well as protecting the target cell against HSV-1 infection (Graphical Abstract). 

Once HSV-1 inhibition and the action mechanism were showed, the relationship between AD-like markers was studied, focusing on the amyloidogenic pathway, and more specifically, studying the effect of NPAuG2-S4 and NPAuG3-S8 in the β-secretase activity and changes in the secreted pattern of Aβ aggregates. Our study reveals that NPAuG2-S4 and NPAuG3-S8 modified AD-like markers by decreasing β-secretase activity altered in the HSV-1 infection, and moreover, reducing those levels to those observed in the non-treated condition. These results are strongly supported by the fact that NPAuG2-S4 and NPAuG3-S8 can recover normal values of the secreted Aβ aggregates produced by HSV-1 infection, either in Aβ40 or in Aβ42. Interestingly, NPAuG3-S8 gold NP crosses the BBB and does not show CNS toxicity at the concentrations and time studied. All these data suggest that the NPAuG3-S8 could be a promising candidate for further studies on the relationship between HSV-1 infection and neurodegenerative diseases (Graphical Abstract).

## Figures and Tables

**Table 1:** Characteristics of the selected NPAuG1-S2, NPAuG2-S4, and NPAuG3-S8.

Nomenclature	Nuclei	Generation	Chargues N	Funtional Group	Molecular Weight (g/mol)
NPAuG1-S2	GOLD	1	2	SULFONATE	421978
NPAuG2-S4	GOLD	2	4	SULFONATE	374956
NPAuG3-S8	GOLD	3	8	SULFONATE	257929

**Table 2:** Brain toxicity assay of NPAuG3-S8.

	PBS			10 H						24 H									48 H					
**mouse ID**	**1**	**2**	**3**	**4**	**5**	**6**	**7**	**8**	**9**	**10**	**11**	**12**	**13**	**14**	**15**	**16**	**17**	**18**	**19**	**20**	**21**	**22**	**23**	**24**
**TELENCEPHALON**																								
Microglia infiltration	0	0	0	0	0	0	0		0	0	0	0	0	0	0	0	0	0	0	0	0	0	0	0
satellitism	0	0	0	0	0	0	0		0	0	0	0	0	0	0	0	0	0	0	0	0	0	0	0
vacuolization	0	0	0	0	0	0	0		0	0	0	0	0	0	0	0	0	0	0	0	0	0	0	0
hemorrhage	0	0	0	0	0	0	0		0	0	0	0	0	0	0	0	0	0	0	0	0	0	0	0
edema	0	0	0	0	0	0	0		0	0	0	0	0	0	0	0	0	0	0	0	0	0	0	0
necrosis	0	0	0	0	0	0	0		0	0	0	0	0	0	0	0	0	0	0	0	0	0	0	0
**DILENCEPHAL**																								
microgliainfiltration			0	0		0	0	0	0			0	0	0	0	0	0	0	0	0	0	0	0	0
satellitism			0	0		0	0	0	0			0	0	0	0	0	0	0	0	0	0	0	0	0
vacuolization			0	0		0	0	0	0			0	0	0	0	0	0	0	0	0	0	0	0	0
hemorrhage			0	0		0	0	0	0			0	0	0	0	0	0	0	0	0	0	0	0	0
edema			0	0		0	0	0	0			0	0	0	0	0	0	0	0	0	0	0	0	0
necrosis			0	0		0	0	0	0			0	0	0	0	0	0	0	0	0	0	0	0	0
**MIDBRAIN**																								
microgliainfiltration		0	0	0	0	0	0	0	0	0	0	0	0	0	0	0	0	0	0	0	0	0	0	0
satellitism		0	0	0	0	0	0	0	0	0	0	0	0	0	0	0	0	0	0	0	0	0	0	0
vacuolization		0	0	0	0	0	0	0	0	0	0	0	0	0	0	0	0	0	0	0	0	0	0	0
hemorrhage		0	0	0	0	0	0	0	0	0	0	0	0	0	0	0	0	0	0	0	0	0	0	0
edema		0	0	0	0	0	0	0	0	0	0	0	0	0	0	0	0	0	0	0	0	0	0	0
necrosis		0	0	0	0	0	0	0	0	0	0	0	0	0	0	0	0	0	0	0	0	0	0	0
**CEREBELLUM**																								
microgliainfiltration	0	0	0				0	0	0	0	0	0		0		0	0	0	0			0	0	
satellitism	0	0	0				0	0	0	0	0	0		0		0	0	0	0			0	0	
vacuolization	0	0	0				0	0	0	0	0	0		0		0	0	0	0			0	0	
hemorrhage	0	0	0				0	0	0	0	0	0		0		0	0	0	0			0	0	
edema	0	0	0				0	0	0	0	0	0		0		0	0	0	0			0	0	
necrosis	0	0	0				0	0	0	0	0	0		0		0	0	0	0			0	0	

The existence of injury in the brain epithelium was evaluated in each biological sample. 0: no change;. These values were added up and determined the level of damage as minimum 1-3, average 4-6, moderate 7-9 and severe 9+.

## References

[1] Kennedy P.G., Rovnak J., Badani H., Cohrs R.J. (2015). A comparison of herpes simplex virus type 1 and varicella-zoster virus latency and reactivation. J. Gen. Virol..

[2] Nicoll M.P., Proenca J.T., Efstathiou S. (2012). The molecular basis of herpes simplex virus latency. FEMS Microbiol. Rev..

[3] Wagner E.K., Bloom D.C. (1997). Experimental investigation of herpes simplex virus latency. Clin. Microbiol. Rev..

[4] Tan I.L., McArthur J.C., Venkatesan A., Nath A. (2012). Atypical manifestations and poor outcome of herpes simplex encephalitis in the immunocompromised. Neurology.

[5] Nicoll M.P., Hann W., Shivkumar M., Harman L.E., Connor V., Coleman H.M., Proenca J.T., Efstathiou S. (2016). The HSV-1 Latency-Associated Transcript Functions to Repress Latent Phase Lytic Gene Expression and Suppress Virus Reactivation from Latently Infected Neurons. PLoS Pathog..

[6] Roizman B., Zhou G. (2015). The 3 facets of regulation of herpes simplex virus gene expression: A critical inquiry. Virology.

[7] Zhou L., Miranda-Saksena M., Saksena N.K. (2013). Viruses and neurodegeneration. Virol. J..

[8] Harris S.A., Harris E.A. (2018). Molecular Mechanisms for Herpes Simplex Virus Type 1 Pathogenesis in Alzheimer’s Disease. Front. Aging Neurosci..

[9] McNamara J., Murray T.A. (2016). Connections Between Herpes Simplex Virus Type 1 and Alzheimer’s Disease Pathogenesis. Curr. Alzheimer Res..

[10] Itzhaki R.F., Lathe R., Balin B.J., Ball M.J., Bearer E.L., Braak H., Bullido M.J., Carter C., Clerici M., Cosby S.L. (2016). Microbes and Alzheimer’s Disease. J. Alzheimers Dis..

[11] Itzhaki R.F., Lathe R. (2018). Herpes Viruses and Senile Dementia: First Population Evidence for a Causal Link. J. Alzheimers Dis..

[12] Readhead B., Haure-Mirande J.V., Funk C.C., Richards M.A., Shannon P., Haroutunian V., Sano M., Liang W.S., Beckmann N.D., Price N.D. (2018). Multiscale Analysis of Independent Alzheimer’s Cohorts Finds Disruption of Molecular, Genetic, and Clinical Networks by Human Herpesvirus. Neuron.

[13] Eimer W.A., Kumar D.K.V., Shanmugam N.K.N., Rodriguez A.S., Mitchell T., Washicosky K.J., Gyorgy B., Breakefield X.O., Tanzi R.E., Moir R.D. (2018). Alzheimer’s Disease-Associated beta-Amyloid Is Rapidly Seeded by Herpesviridae to Protect against Brain Infection. Neuron.

[14] Gatz M., Reynolds C.A., Fratiglioni L., Johansson B., Mortimer J.A., Berg S., Fiske A., Pedersen N.L. (2006). Role of genes and environments for explaining Alzheimer disease. Arch. Gen. Psychiatry.

[15] Mancuso R., Baglio F., Agostini S., Cabinio M., Lagana M.M., Hernis A., Margaritella N., Guerini F.R., Zanzottera M., Nemni R. (2014). Relationship between herpes simplex virus-1-specific antibody titers and cortical brain damage in Alzheimer’s disease and amnestic mild cognitive impairment. Front. Aging Neurosci..

[16] Lovheim H., Gilthorpe J., Adolfsson R., Nilsson L.G., Elgh F. (2015). Reactivated herpes simplex infection increases the risk of Alzheimer’s disease. Alzheimers Dement..

[17] Tanzi R.E. (2012). The genetics of Alzheimer disease. Cold Spring Harb. Perspect. Med..

[18] Masters C.L., Bateman R., Blennow K., Rowe C.C., Sperling R.A., Cummings J.L. (2015). Alzheimer’s disease. Nat. Rev. Dis. Primers.

[19] Andrew R.J., Kellett K.A., Thinakaran G., Hooper N.M. (2016). A Greek Tragedy: The Growing Complexity of Alzheimer Amyloid Precursor Protein Proteolysis. J. Biol. Chem..

[20] Lakey-Beitia J., Berrocal R., Rao K.S., Durant A.A. (2015). Polyphenols as therapeutic molecules in Alzheimer’s disease through modulating amyloid pathways. Mol. Neurobiol..

[21] Marlow L., Cain M., Pappolla M.A., Sambamurti K. (2003). Beta-secretase processing of the Alzheimer’s amyloid protein precursor (APP). J. Mol. Neurosci..

[22] Santana S., Bullido M.J., Recuero M., Valdivieso F., Aldudo J. (2012). Herpes simplex virus type I induces an incomplete autophagic response in human neuroblastoma cells. J. Alzheimers Dis..

[23] Verbeek M.M., Otte-Holler I., Fransen J.A., de Waal R.M. (2002). Accumulation of the amyloid-beta precursor protein in multivesicular body-like organelles. J. Histochem. Cytochem..

[24] Santana S., Recuero M., Bullido M.J., Valdivieso F., Aldudo J. (2012). Herpes simplex virus type I induces the accumulation of intracellular beta-amyloid in autophagic compartments and the inhibition of the non-amyloidogenic pathway in human neuroblastoma cells. Neurobiol. Aging.

[25] Piacentini R., Puma D.D.L., Ripoli C., Marcocci M.E., de Chiara G., Garaci E., Palamara A.T., Grassi C. (2015). Herpes Simplex Virus type-1 infection induces synaptic dysfunction in cultured cortical neurons via GSK-3 activation and intraneuronal amyloid-beta protein accumulation. Sci. Rep..

[26] Alvarez G., Aldudo J., Alonso M., Santana S., Valdivieso F. (2012). Herpes simplex virus type 1 induces nuclear accumulation of hyperphosphorylated tau in neuronal cells. J. Neurosci. Res..

[27] Lin W.R., Wozniak M.A., Cooper R.J., Wilcock G.K., Itzhaki R.F. (2002). Herpesviruses in brain and Alzheimer’s disease. J. Pathol..

[28] Coen D.M., Schaffer P.A. (2003). Antiherpesvirus drugs: A promising spectrum of new drugs and drug targets. Nat. Rev. Drug Discov..

[29] Superti F., Ammendolia M.G., Marchetti M. (2008). New advances in anti-HSV chemotherapy. Curr. Med. Chem..

[30] Burrel S., Boutolleau D., Azar G., Doan S., Deback C., Cochereau I., Agut H., Gabison E.E. (2013). Phenotypic and genotypic characterization of acyclovir-resistant corneal HSV-1 isolates from immunocompetent patients with recurrent herpetic keratitis. J. Clin. Virol..

[31] van Velzen M., van Loenen F.B., Meesters R.J., de Graaf M., Remeijer L., Luider T.M., Osterhaus A.D., Verjans G.M. (2012). Latent acyclovir-resistant herpes simplex virus type 1 in trigeminal ganglia of immunocompetent individuals. J. Infect. Dis..

[32] Ljungman P., Ellis M.N., Hackman R.C., Shepp D.H., Meyers J.D. (1990). Acyclovir-resistant herpes simplex virus causing pneumonia after marrow transplantation. J. Infect. Dis..

[33] Briz V., Sepulveda-Crespo D., Diniz A.R., Borrego P., Rodes B., de la Mata F.J., Gomez R., Taveira N., Munoz-Fernandez M.A. (2015). Development of water-soluble polyanionic carbosilane dendrimers as novel and highly potent topical anti-HIV-2 microbicides. Nanoscale.

[34] Guerrero-Beltran C., Cena-Diez R., Sepulveda-Crespo D., de la Mata J., Gomez R., Leal M., Munoz-Fernandez M.A., Jimenez J.L. (2017). Carbosilane dendrons with fatty acids at the core as a new potential microbicide against HSV-2/HIV-1 co-infection. Nanoscale.

[35] Cena-Diez R., Vacas-Cordoba E., Garcia-Broncano P., de la Mata F.J., Gomez R., Maly M., Munoz-Fernandez M.A. (2016). Prevention of vaginal and rectal herpes simplex virus type 2 transmission in mice: Mechanism of antiviral action. Int. J. Nanomed..

[36] Garcia-Broncano P., Cena-Diez R., de la Mata F.J., Gomez R., Resino S., Munoz-Fernandez M.A. (2017). Efficacy of carbosilane dendrimers with an antiretroviral combination against HIV-1 in the presence of semen-derived enhancer of viral infection. Eur. J. Pharmacol..

[37] Cena-Diez R., Garcia-Broncano P., de la Mata F.J., Gomez R., Munoz-Fernandez M.A. (2016). Efficacy of HIV antiviral polyanionic carbosilane dendrimer G2-S16 in the presence of semen. Int. J. Nanomed..

[38] Vacas-Cordoba E., Maly M., de la Mata F.J., Gomez R., Pion M., Munoz-Fernandez M.A. (2016). Antiviral mechanism of polyanionic carbosilane dendrimers against HIV-1. Int. J. Nanomed..

[39] Bermejo J.F., Ortega P., Chonco L., Eritja R., Samaniego R., Mullner M., de Jesus E., de la Mata F.J., Flores J.C., Gomez R. (2007). Water-soluble carbosilane dendrimers: Synthesis biocompatibility and complexation with oligonucleotides; evaluation for medical applications. Chemistry.

[40] Sepulveda-Crespo D., Sanchez-Rodriguez J., Serramia M.J., Gomez R., de la Mata F.J., Jimenez J.L., Munoz-Fernandez M.A. (2015). Triple combination of carbosilane dendrimers, tenofovir and maraviroc as potential microbicide to prevent HIV-1 sexual transmission. Nanomedicine.

[41] Sepulveda-Crespo D., Serramia M.J., Tager A.M., Vrbanac V., Gomez R., de la Mata F.J., Jimenez J.L., Munoz-Fernandez M.A. (2015). Prevention vaginally of HIV-1 transmission in humanized BLT mice and mode of antiviral action of polyanionic carbosilane dendrimer G2-S16. Nanomedicine.

[42] Ghasemi F., Hormozi-Nezhad M.R., Mahmoudi M. (2018). Label-free detection of beta-amyloid peptides (Abeta40 and Abeta42): A colorimetric sensor array for plasma monitoring of Alzheimer’s disease. Nanoscale.

[43] Zheng K., Liu Q., Wang S., Ren Z., Kitazato K., Yang D., Wang Y. (2018). HSV-1-encoded microRNA miR-H1 targets Ubr1 to promote accumulation of neurodegeneration-associated protein. Virus Genes.

